# Supplementary figures and images for: Impact of deep learning and post-processing algorithms performances on biodiversity metrics assessed on videos
Source: PLoS One. 2025 Aug 11;20(8):e0327577. doi: 10.1371/journal.pone.0327577 (PMC12338835; doi:10.1371/journal.pone.0327577)

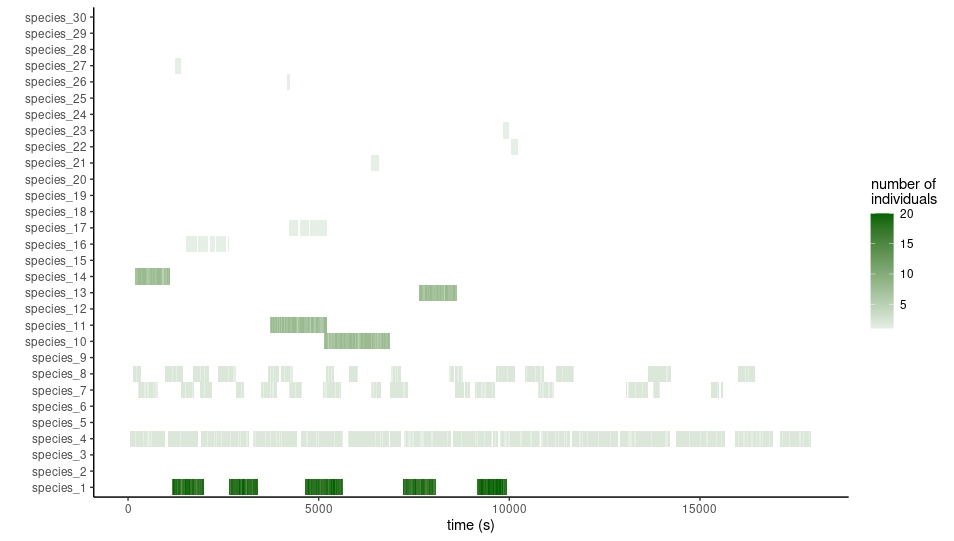

Supplement: S1 Fig — The number of individuals of each species is illustrated with shades of green (white slots indicate absence of species). (TIF) [file pone.0327577.s001.tif]

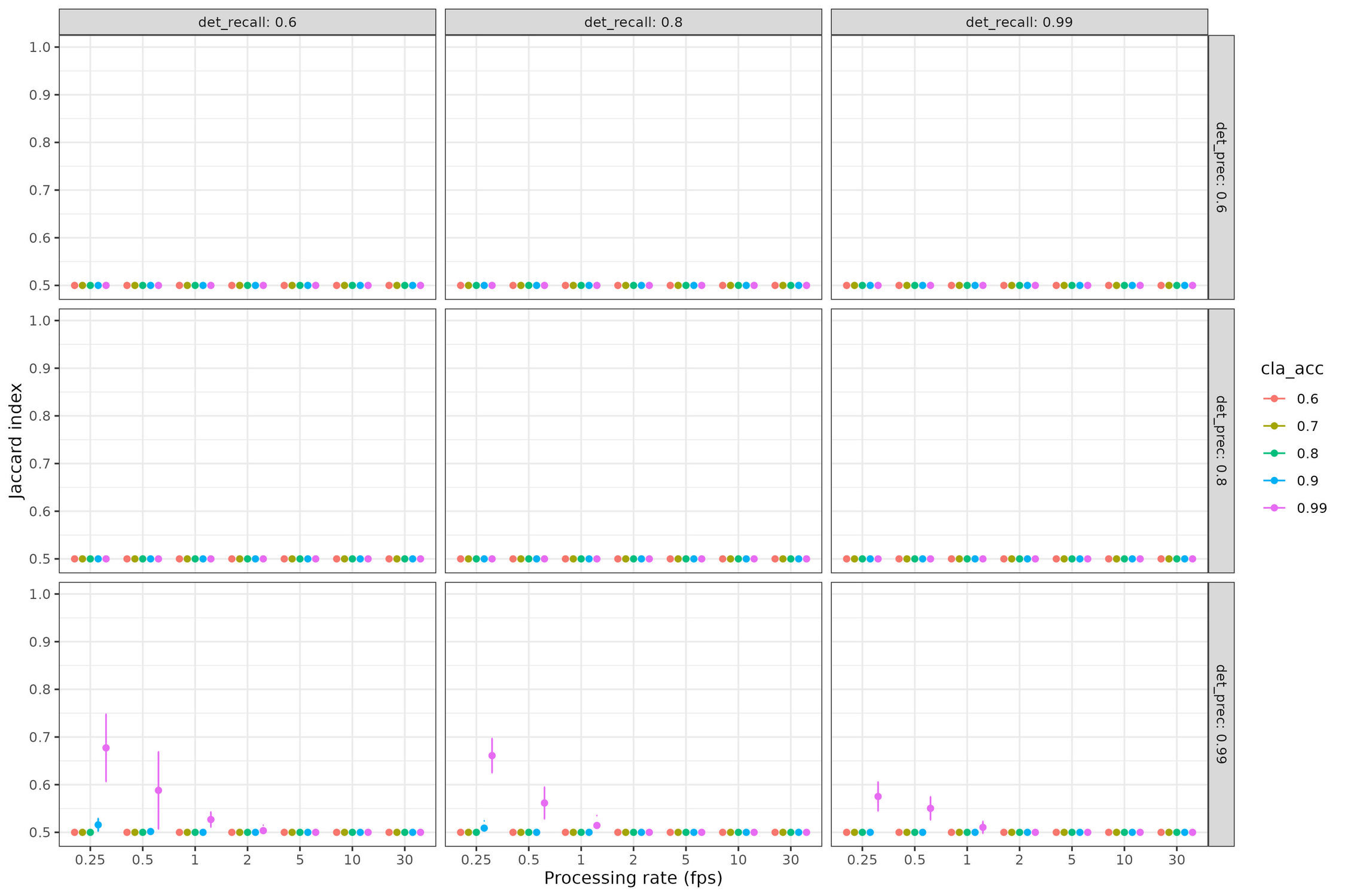

Supplement: S2 Fig — Estimation of the species composition as a function of video processing rate (fps, frame per second) for 45 automated analysis models resulting from 9 detection models, with their respective recall (“det_recall” – columns) and precision (“det_prec” – rows) performances and 5 classification models (accuracy (“cla_acc”) as colors). Each dot represents the average over the 10 simulations with corresponding standard error as vertical bars. The Jaccard similarity index was used to measure the difference in composition of species present on the videos and the composition estimated after automated processing using algorithms. (TIF) [file pone.0327577.s003.tif]

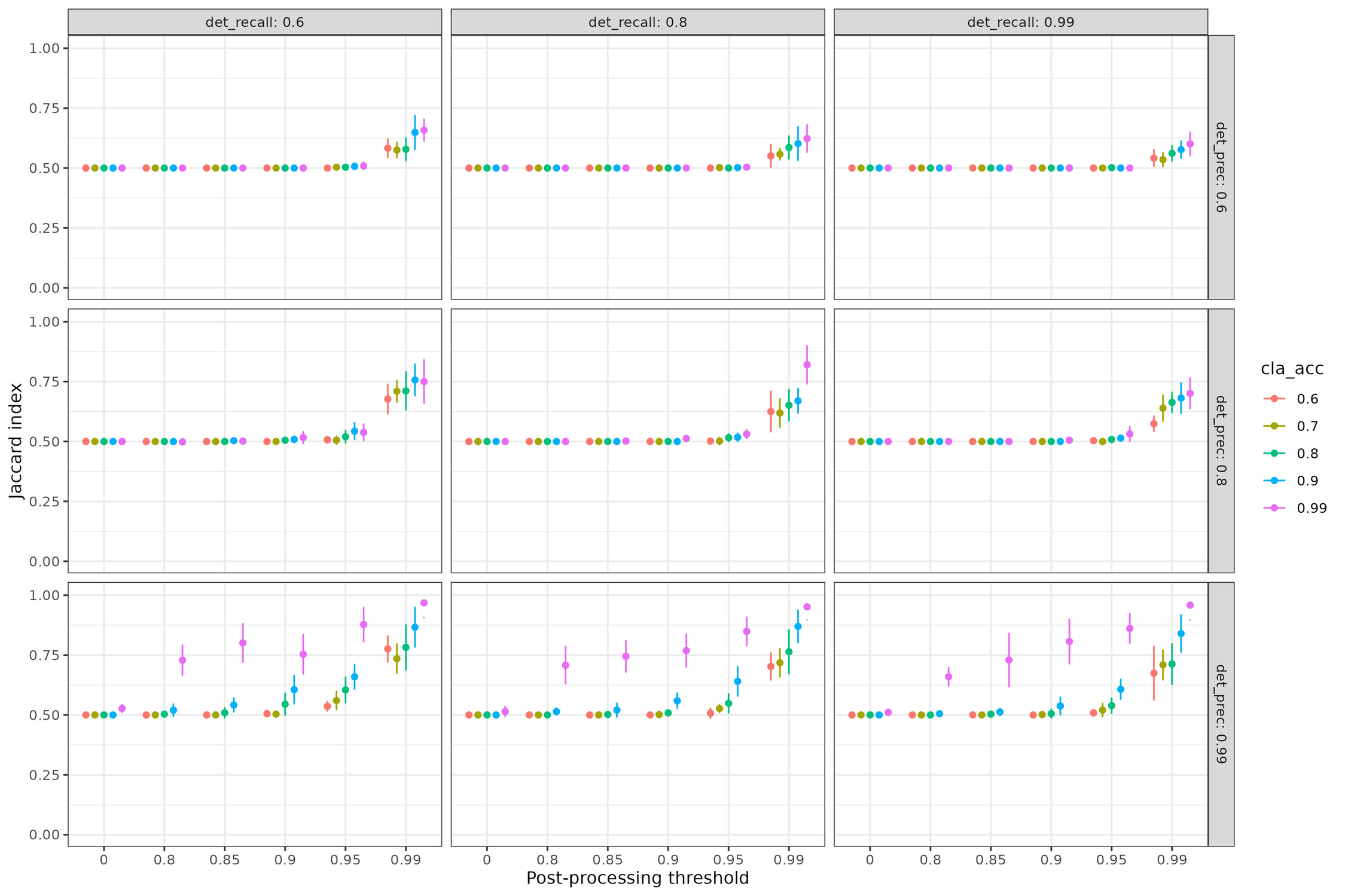

Supplement: S3 Fig — Estimation of the species composition as a function of post-processing threshold for 45 automated analysis models resulting from 9 detection models, with their respective recall (“det_recall” – columns) and precision (“det_prec” – rows) performances and 5 classification models (accuracy (“cla_acc”) as colors) for 1 frame per second processing rate. Each dot represents the average over the 10 simulations and the error bars are shown. Each panel gathers results for a detection algorithm (recall in column and precision in row) and a classification algorithm (color corresponding to its accuracy). Post-processing applies a confidence threshold to outputs of the identification models, discarding those with the lowest confidence scores to minimize misidentifications. The Jaccard similarity index was used to measure the difference in composition of species present on the videos and the composition estimated after automated processing using algorithms. (TIF) [file pone.0327577.s004.tif]
