# Supplementary material for: Impact of deep learning and post-processing algorithms performances on biodiversity metrics assessed on videos
Source: PLoS One. 2025 Aug 11;20(8):e0327577. doi: 10.1371/journal.pone.0327577 (PMC12338835; doi:10.1371/journal.pone.0327577)
Supplement: S1 Table — Values are the number of simulations (out of 10) yielding 12, 13, 14, or 15 detectable species (i.e., at least 1 individual present on at least one frame). For each of the 7 processing rates (frames per second). (DOCX) [file pone.0327577.s002.docx]

| processing rate (fps) | Number of species detectable | | | |
| --- | --- | --- | --- | --- |
|  | 12 | 13 | 14 | 15 |
| 0.25 | 0 | 1 | 5 | 4 |
| 0.5 | 0 | 0 | 2 | 8 |
| 1 | 0 | 0 | 0 | 10 |
| 2 | 0 | 0 | 0 | 10 |
| 5 | 0 | 0 | 0 | 10 |
| 10 | 0 | 0 | 0 | 10 |
| 30 | 0 | 0 | 0 | 10 |
