# Supplementary material for: Impact of deep learning and post-processing algorithms performances on biodiversity metrics assessed on videos
Source: PLoS One. 2025 Aug 11;20(8):e0327577. doi: 10.1371/journal.pone.0327577 (PMC12338835; doi:10.1371/journal.pone.0327577)
Supplement: Attachment — Submitted filename: authors_reponse_auresp_2.docx [file pone.0327577.s006.docx]

We have carefully revised the manuscript to correct the grammatical errors, as suggested by the reviewer. For an easy reading of the following answers, the authors’ responses are in blue font.

**Comments to the Author**

Reviewer #1:

Thank you for your careful reading and helpful corrections of grammatical errors, which improved the clarity of the manuscript.

Line 23 – video based surveys are

Done

Line 27/80 – “However, the impacts on models' performances and usage on assessment of biodiversity metrics on videos remains unknown.” Or better say “is yet to be assessed” to reflect wording in the into.

Done

Line 29 – The sentence is long and unclear – either say “14,406 simulated automated processing pipelines” or reword to “Using simulated remote videos of fish communities processed with 14,406 simulated automated pipelines, …”

Done

Line 43 – consistently tracking (swap order)

Done

Line 55 – change developing to “development of”

Done

Line 79 – “false positives (regions of image without any individuals but detected as containing one).”

Done

Line 97 – “is yet to be assessed”

Done

Line 119 – Intro ends a bit abruptly. Add one line at end of intro to summarise how. E.g. “We achieve this using simulated remote videos of fish communities and simulated 14,406 automated 32 processing pipelines, and calculating error rates and biodiversity metrics.”

Done

Line 116 –“The number of replicates was limited to 10 due to high computational cost.” Better say “to balance computational cost with reproducibility”, to show your results were reproducible even with 10 repeats.

Done
